# Diseases of Atlanta, Georgia

**Published:** 1856-12

**Authors:** J. G. Westmoreland

**Affiliations:** Atlanta, Georgia


### ARTICLE II.

*Diseases of Atlanta, Georgia.* By J. G. WESTMORELAND, M. D.

Prior to the first of May, 1853, the time I came to Atlanta, there had been, for two years or more, a prevailing epidemic of Typhoid fever. During its prevalence I occasionally visited the place, and from my observation no unusual malignancy seemed to be manifested in the Fever. Although the subjects of its attack were numerous, much less mortality attended it than is sometimes found.

This was the more to be wondered at, since at that time those causes which generally induce malignancy in epidemic diseases of any kind, were to be found in abundance here. It is notorious that the rapid settlement of any place, where numerous improvements are going on at the same time, by which the forest-growth is destroyed, and the surface of the earth disturbed extensively, is a fruitful source of disease. This might have favored the appearance and extensive ravages of the epidemic, but did not seem in this disease to add to its violence.

The sufferers from other diseases, in many instances, were not so fortunate.

Measles, which about this time, prevailed also extensively, was attended with unusually violent symptoms. Obstinate, and often uncontrollable Diarrhœa, coming on as the sequelæ, or as a complication in the disease, was the cause of considerable mortality during the warm season. In some instances Dysenteric symptoms would be the first to show disturbance of the bowels, but would usually give place to those of malignant Diarrhœa. From the information derived from some practitioners, the former name would be more appropriate, yet doubtless the inflammation of the mucous surface of the bowels was more extensive than is found in ordinary Dysentery.

In fact, since my observation of disease in Atlanta, and from the report of others even before, there has not been an epidemic of Malignant Dysentery in the city at any time. It is true, I have witnessed a number of mild cases occurring in the same neighborhood at the same time, but they differed materially from those usually termed epidemic. They were generally controlled readily by the ordinary means.

The city has rarely been exempt from Measles from that time to the present; but has usually been accompanied with less fatal local inflammatory derangement. Bronchial inflammation, extending sometimes to the parenchyma is the common difficulty, and rarely proves fatal. Varicella occasionally makes its appearance, and during the Small-pox panic last Spring, both it and Measles were occasionally mistaken for Variola.

These mistakes occurred, of course, with those out of the profession; but to gratify the propensity which some have, in a high degree, to be the first to tell alarming news, there were those who would circulate these false reports as coming from a physician, greatly to the disturbance of the citizens.

Following Measles came Roseola; and a peculiarity in disease I had never witnessed before was manifested. All those who had been affected were particularly subject to the eruption. So much so, that I do not recollect a single instance of the latter which had not been the subject of the former. This secondary eruption occurred often several months after the attack of Measles.

Scarlatina, in the malignant and milder forms, has been found occurring sporadically most of the time for the last three years. Cases frequently occur simultaneously in remote parts of the city from each other, and in some instances, attack only one in a family or even in the neighborhood. Occasionally one of these isolated cases proves to be of the most malignant character, running its course rapidly to a fatal termination. At no period since my residence in the city have I

known the disease to prevail extensively, nor to spread from those affected, as if by contagion. A remarkable instance of the attack of Rubeola and Scarlatina, in the same individual, was observed last spring by a physician of this place. It seems, from the history of the case, that the subject, a boy eight or ten years old, had been exposed to the contagion of Measles, and a few days afterwards, perhaps only two or three, he was attacked with Scarlet fever, which ran the usual course of Scarlatina Anginosa, and while convalescent the eruption of Measles made its appearance with the usual symptoms of that disease. I was kindly invited by the attending physician to witness this anomalous succession of diseases, and found the fading eruption of Rubeola with the usually accompanying cough, from bronchial irritation, while the marks of counter-irritation produced by the treatment for inflamed tonsils in the former disease were still visible. I did not examine the patient while either form of the eruption was distinct, but learned from his physician that they exhibited all the distinctive features of the two diseases.

Remittent and Intermittent Fevers do not occur very frequently, yet considering the absence of those causes which commonly produce Malarious Fever, it is surprising that the disease should be found at all. It is true that perhaps half the cases that are met with here, are contracted in malarious districts; at the same time there are those that receive this unknown subtle poison in the locality. I have seen cases in the heart of the city, on White-Hall Street, the subjects of which had not been out of town for months. I have also seen several cases near each other, on the skirts of the city, in the neighborhood of a small marshy branch imperfectly drained. These cases were few and of a very mild character, proving as well by the symptoms as by the topography of the vicinity, that the effluvia was limited in quantity as well as in virulence. Typhoid fever, though occurring only occasionally and in milder form since the epidemic of 1851–2, is still in existence amongst us; and that individual is peculiarly unfortunate, who contracts this disease in the midst of cases of Remittent Fever. Not that the diseases themselves have any controlling influence, one over the other, but that where Remittent Fever is the prevailing disease, there is great danger of mistaking Typhoid fever for it, when the latter is met with. An error of this kind may cost the sick man his life. There is no generally recognized pathognomonic symptom of Typhoid Fever; and the usually observed symptoms may be so nearly the same, that the practitioner may be easily deceived, especially when the prevailing disease is of the remittent character. Let this error be counted by the physician, and let the abortive treatment usually applied to Remittent Fever be adopted and persisted in, and the tenure of the patient's earthly existence will be of short duration. We want some symptom by which we can determine the existence of this Fever within the first two or three days of the disease. That symptom we have.—Though not generally recognized, it nevertheless exists. The evidences of Typhoid Fever, are pain in the region of the medulla oblongata, tenderness on pressure just below the occiput, in the depression opposite the first cervical vertebra posteriorly, and pressure opposite the same vertebra laterally, alternately with the thumb and finger placed on opposite sides, so as to communicate a gentle motion to the bone together with absence of lumber and dorsal pain and tenderness. I can see no good reason why locality can have any influence upon the disease, yet it has occurred in my practice less frequently here than elsewhere.

